# Evaluation of Changes in Macular Perfusion Detected by Optical Coherence Tomography Angiography following 3 Intravitreal Monthly Bevacizumab Injections for Diabetic Macular Edema in the IMPACT Study

**DOI:** 10.1155/2020/5814165

**Published:** 2020-04-27

**Authors:** Ayman G. Elnahry, Ahmed A. Abdel-Kader, Karim A. Raafat, Khaled Elrakhawy

**Affiliations:** Department of Ophthalmology, Faculty of Medicine, Cairo University, Cairo, Egypt

## Abstract

**Objective:**

To evaluate macular perfusion changes following intravitreal bevacizumab injections for diabetic macular edema (DME) using spectral domain optical coherence tomography angiography (SD-OCTA).

**Methods:**

This study was a prospective noncomparative interventional case series. Treatment naïve patients with DME underwent full ophthalmological examination and SD-OCTA scanning at baseline and after 3 intravitreal bevacizumab injections. Both the 6 × 6 and 3 × 3 mm macular scan protocols were used. Pretreatment and posttreatment OCTA images were automatically aligned using a commercially available retina alignment software (i2k Align Retina software); then the fractal dimension (FD), vascular density (VD), and skeleton VD changes were obtained at the full retinal thickness (Full) and superficial (SCP) and deep (DCP) capillary plexuses after processing images using a semiautomated program. The foveal avascular zone (FAZ) was manually measured and FD was calculated using the FracLac plugin of ImageJ.

**Results:**

Forty eyes of 26 patients were included. Following injections, there were an 8.1% increase in FAZ, 1.3% decrease in FD-Full and FD-SCP, 1.9% decrease in FD-DCP, 8% decrease in VD-Full, 9.1% decrease in VD-SCP, 10.6% decrease in VD-DCP, 13.3% decrease in skeleton VD-Full, 12.5% decrease in skeleton VD-SCP, and 16.3% decrease in skeleton VD-DCP in the 6 × 6 mm macular area and a 2.6% decrease in FD-Full, 3.4% decrease in FD-SCP, 11.5% decrease in VD-Full, 14.3% decrease in VD-SCP, and 25.1% decrease in skeleton VD-SCP in the 3 × 3 mm macular area which were all statistically significant (*p* < 0.05). Using univariate and multivariate analysis, the pretreatment FD, VD, and skeleton VD at each capillary layer significantly negatively correlated with the change in FD, VD, and skeleton VD at the corresponding capillary layer, respectively (*p* < 0.05).

**Conclusion:**

OCTA is a useful noninvasive tool for quantitative evaluation of macular perfusion changes following DME treatment. This trial is registered with NCT03246152.

## 1. Background

The prevalence of diabetes mellitus (DM) has progressively increased worldwide in recent years and is expected to grow to 430 million by 2030 [1]. In the United States, an estimated 29% of adults with DM have diabetic retinopathy (DR) and 3% have diabetic macular edema (DME) [2], with DM being the leading cause of blindness in people between the ages of 20 and 74 [3]. In Egypt, many patients are found to have DM and in a study of Egyptian patients with DM, diabetic retinopathy was present in 42% and legal blindness in 5% [4]. This was higher than the worldwide prevalence rate of DR in diabetic patients which is estimated at 34.6% [3].

DME is the commonest cause of loss of vison in diabetics that occurs due to accumulation of fluid in the central macular region following dysfunction of the blood retinal barrier [5]. This occurs due to release of various inflammatory cytokines and growth factors, most importantly vascular endothelial growth factor (VEGF), following chronic hyperglycemia [6]. Several treatment modalities are available for DME, including the intravitreal injection of anti-VEGF drugs such as off-label intravitreal bevacizumab injections, which are also used in the treatment of macular edema associated with various other retinal conditions [7–11]. Because the therapeutic effect of these drugs is only temporary, however, long-term treatment is often required which raises concerns for worsening of macular ischemia and visual function due to chronic suppression of VEGF that could result in increased capillary dropout and inhibition of reperfusion of occluded capillaries [12, 13].

Most of the studies evaluating the effect of repeated intravitreal anti-VEGF injections on the macular perfusion status in diabetics have relied on qualitative analysis of perfusion using fluorescein angiography (FA) [14–16]. Optical coherence tomography angiography (OCTA) is a relatively new noninvasive modality capable of analyzing the different retinal capillary layers quantitatively at a higher resolution compared to conventional FA [17]. The Evaluation of the Effect of Intravitreal Injections of Anti-VEGF on Macular Perfusion in Diabetic Patients using OCTA (IMPACT) study was a prospective noncomparative interventional case series done to evaluate the effect of repeated intravitreal bevacizumab injections on the macular perfusion of diabetic patients using OCTA.

## 2. Methods

### 2.1. Study Purpose and Design

The IMPACT study was a prospective noncomparative interventional case series done at Cairo University Hospital to evaluate the effect of three intravitreal monthly bevacizumab injections on the macular perfusion of diabetic patients with diabetic macular edema using OCTA.

### 2.2. Ethical Approval and Study Registration

Study approval was obtained from Cairo University Research Ethics Committee, and the study followed the tenets of the Declaration of Helsinki. A clinical trial registration was done at ClinicalTrials.gov on August 11, 2017, before beginning patient recruitment (study identifier: NCT03246152). Written informed consent was obtained from all study participants before inclusion in the study.

### 2.3. Study Outcome Measures

The primary outcome measures of the study were the change in the FAZ area, fractal dimension, vascular density, and skeletonized vascular density following the injections. The secondary outcome measure was the change in corrected distance visual acuity following injections. Although the change in microaneurysms appearance and number was a predefined secondary outcome measure in the study, this analysis was later abandoned due to the low sensitivity of OCTA in detecting microaneurysms.

### 2.4. Recruitment of Patients

Diabetic patients attending the retina clinic of Cairo University Hospital between October 2017 and September 2018 underwent comprehensive history taking and ophthalmological examination including corrected distance visual acuity (CDVA), intraocular pressure (IOP) measurement, anterior segment, and posterior segment examination. Diabetic patients who were 18 years or older and complaining of blurring of vision with a CDVA of less than 20/30 and evidence of macular edema on clinical examination underwent spectral domain optical coherence tomography (SD-OCT) (Optovue, Inc., Fremont, California, USA) and FA (TRC 50DX, Topcon, Tokyo, Japan). Patients with center involving diabetic macular edema on SD-OCT with a central macular thickness (CMT) of more than or equal to 275 *μ*m were recruited for the study. Exclusion criteria included concomitant ocular conditions that may affect retinal perfusion such as branch or central retinal vein occlusion and uveitis, history of vitreoretinal surgeries, history of previous intravitreal injections or macular laser treatment for diabetic macular edema, high myopia (refraction ≤ −6 D), presence of an epiretinal membrane or vitreomacular traction, significant media opacities, uncontrolled glaucoma, and systemic thromboembolic events within the last 6 months.

### 2.5. Recruited Patients

Patients who were enrolled in the study then underwent a baseline macular SD-OCTA scan (Optovue, Inc., Fremont, California, USA) before starting bevacizumab injections. Each patient then underwent 3 consecutive monthly intravitreal bevacizumab injections.

Patients were instructed to follow up closely with an internist to tightly control their blood sugar, blood lipids, and blood pressure during the study period, and an HbA1C measurement was obtained at the time of the last injection to determine the level of blood sugar control during the treatment period.

Patients were followed up 1 day, 1 week, and 1 month at the retina clinic following the injections and one month following the third intravitreal injection a final full ophthalmological examination was performed and documented and a follow-up OCTA scan was done using the same pretreatment protocol.

### 2.6. Acquisition of OCTA Images

The AngioVue software of the RTVue XR Avanti SD-OCTA (Optovue, Inc, Fremont, CA), which uses a split-spectrum amplitude decorrelation angiography (SSADA) algorithm to improve signal-to-noise ratio, was used. The device operates at a wavelength of approximately 840 nm and obtains 70,000 A-scans per second. The OCTA volume scan consisted of 2 repeated B-scans from 304 sequentially uniformly spaced locations. Each B-scan consisted of 304 A-scans with an axial resolution of about 5 *μ*m. The resultant three-dimensional OCTA flow data in the full retinal thickness (Full) and the superficial (SCP) and deep (DCP) capillary plexuses were projected into two-dimensional en-face OCTA images that were used in the study. Automated segmentation was performed by the machine as follows: the full retinal thickness (Full) slab was segmented from the internal limiting membrane (ILM) to 9 *μ*m below the outer plexiform layer- (OPL-) outer nuclear layer (ONL) junction, while the SCP was segmented from the ILM to 9 *μ*m above the inner plexiform layer- (IPL-) inner nuclear layer (INL) junction, and the DCP was segmented from 9 *μ*m above the IPL-INL junction to 9 *μ*m below the OPL-ONL junction. Two scan protocols, the 6 × 6 mm and the 3 × 3 mm macular scans, which were centered on the fovea, were used. The 6 × 6 mm macular scan protocol was done for all included patients, while the 3 × 3 mm macular scan protocol was done for some of the included patients. Both scanning protocols were used since the 6 × 6 mm scan allowed imaging of the perifoveal capillary network, while the 3 × 3 mm scan provided a higher resolution of the foveal avascular zone (FAZ) and the parafoveal capillary network.

To ensure the acquisition of only high-quality images, scans were repeated if there was an insufficient signal strength index (SSI; <5), presence of 1 or more blink artifacts, poor fixation resulting in motion or doubling artifacts, areas of localized signal loss from media opacity obscuring the vasculature, or major segmentation errors. If following repeated scans one of the aforementioned criteria persisted, the patient was excluded from the study. Minor segmentation errors were corrected manually with the built-in machine software using the corresponding structural OCT B-scans as a guide for the placement of the 2 parallel segmentation lines at the appropriate depths.

### 2.7. Image Alignment and Registration

**Figure 1:**
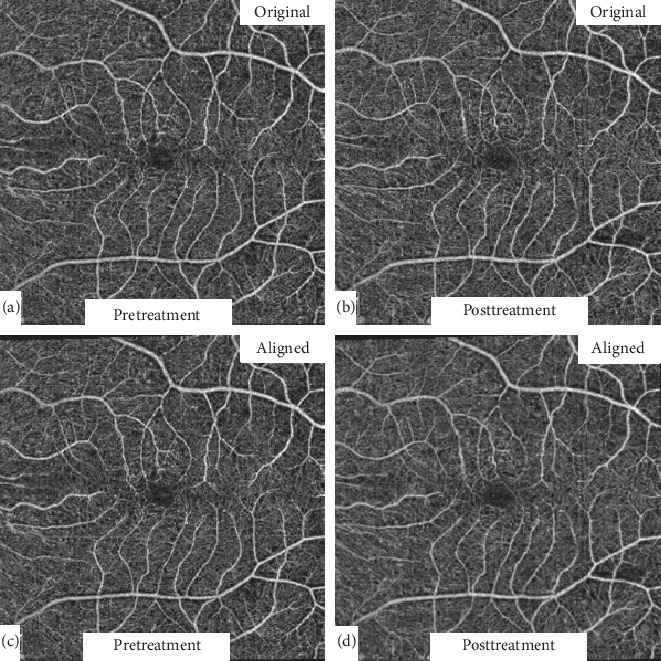
Automated image alignment. Original 6 × 6 mm pre- and posttreatment images (a and b) were automatically registered and aligned (c and d) using a commercially available retina alignment software to allow comparison of only areas common to both images.

Posttreatment OCTA images (following 3 injections) of all patients were automatically registered and aligned with the corresponding pretreatment images at each capillary layer using a commercially available retina alignment software (i2k Align Retina software (DualAlign, LLC, Clifton Park, NY)) [18]. The intersection option of the software was used to align the images which allowed comparison of only areas common to both pre- and posttreatment images (Figure 1). This was necessary since the macular area imaged in the pre- and posttreatment scans was not automatically registered during image acquisition by the built-in machine software.

### 2.8. Measurement of the Foveal Avascular Zone (FAZ) Area (Primary Outcome Measure)

**Figure 2:**
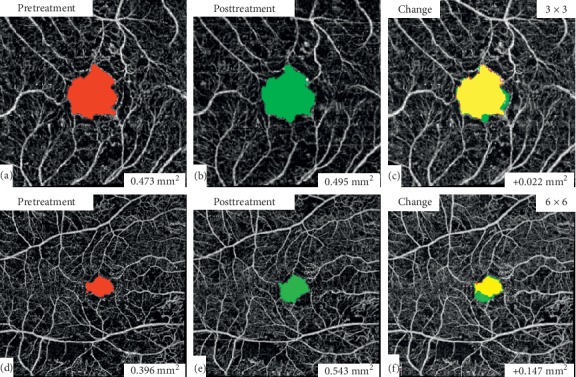
FAZ area measurement. The FAZ area was measured manually using ImageJ for the pre- and posttreatment 3 × 3 mm (a and b) and 6 × 6 mm (d and e) macular scan images. FAZ area change between pre- and posttreatment images was then calculated (c and f).

The FAZ area was measured for each patient before and after the 3 injections and compared. The pre- and posttreatment full retinal thickness images were used, and measurements were performed manually in all patients by a single investigator (A.G.E.) using the freehand tool of ImageJ (National Institutes of Health, Bethesda, Maryland, USA) (Figure 2). Images were scaled to millimeter units before measurements; then three measurements were obtained for each image and an average value was calculated. Pre- and posttreatment FAZ area measurements were compared for each patient and the FAZ change was documented.

### 2.9. Measurement of the Vascular Density (VD) and the Fractal Dimension (FD) (Primary Outcome Measure)

**Figure 3:**
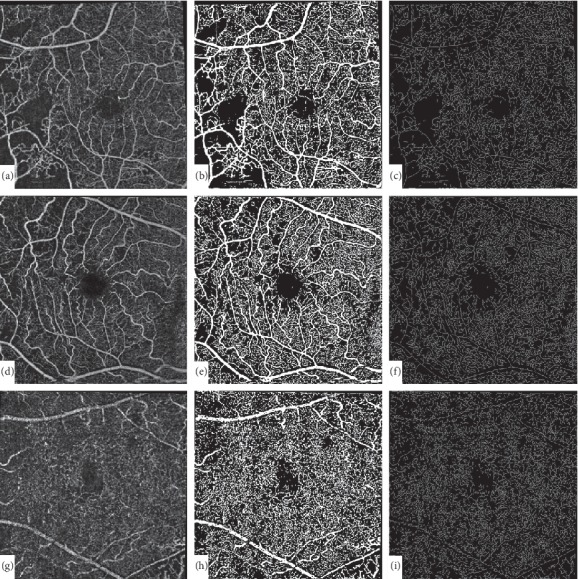
Image processing technique. En-face OCTA images (a, d, and g) were converted into a binary image (b, e, and h) by using a combined method consisting of a global threshold, hessian filter, and adaptive threshold in MATLAB. Skeletonized images were created by deleting the pixels in the outer boundary of the binarized, white-pixelated vessels until only 1 pixel remained along the width of the vessels (c, f, and i).

In order to quantify the changes in VD and FD following treatment, all aligned pre- and posttreatment images were semiautomatically processed and quantified using a previously described and validated method and then compared at each capillary layer [19, 20]. Briefly, processing and quantification of images was done as follows: en-face OCTA images were converted into a binary image by using a combined 3-way method consisting of a global threshold, hessian filter, and adaptive threshold in MATLAB (MathWorks, Inc., Natick, MA, USA). First, an area within the foveal avascular zone was manually selected 3 times with a fixed radius circle (50 pixels) to establish a baseline signal-to-noise ratio for the global thresholding step (Figures 3(a), 3(d), and 3(g)). This was the only manually performed step in the process and was done by the same investigator for all images for consistency (A.G.E). The image was then processed using a top-hat filter. This image was then automatically processed to create two separate binarized images: one created with the application of hessian filter, and another created through median adaptive thresholding. Finally, the resulting two binarized vessel maps obtained were combined to form the final binarized image (Figures 3(b), 3(e), and 3(h)). Only pixels that were detected using both the hessian filter and adaptive thresholding were included in the final binarized image. After acquiring the binary image, VD was calculated as the total image area occupied by the detected OCTA signal (white pixels in the binarized image) compared to total area of retina (total number of pixels in image). Skeletonized images were also created by deleting the pixels in the outer boundary of the binarized, white-pixelated vessels until only 1 pixel remained along the width of the vessels (Figures 3(c), 3(f), and 3(i)). VD was calculated for the skeletonized images as described for the binarized images.

Fractal dimension (FD) which provides a global index for the branching complexity of the retinal capillary network was calculated for the skeletonized images using the FracLac plugin of ImageJ with the box-counting method. Values were calculated for all pretreatment and posttreatment images and compared for each patient.

### 2.10. Statistical Analysis

Data were coded and entered using the Statistical Package for the Social Sciences (SPSS) version 25 (IBM, Chicago, IL, USA). Data was summarized using mean, standard deviation, median, minimum, and maximum in quantitative data and using frequency (count) and relative frequency (percentage) for categorical data. Comparisons between quantitative variables were done using the nonparametric Kruskal-Wallis and Mann-Whitney tests. For comparison of serial measurements within same patients, paired *t*-test was used in normally distributed quantitative variables, while nonparametric Wilcoxon signed rank test was used for nonnormally distributed quantitative variables. Correlations between quantitative variables were done using Pearson or Spearman correlation coefficient. Linear regression analysis was done to predict changes in different parameters using baseline data. *p* values less than 0.05 were considered as statistically significant.

## 3. Results

### 3.1. Patient Demographics

A total of 52 eyes of 31 diabetic patients were found to be eligible and were enrolled in the study in the period between October 2017 and September 2018. Six eyes of 3 patients were lost to follow up or did not adhere to the study protocol and so were excluded from the study, while 6 eyes of 4 patients were excluded due to insufficient OCTA image quality in either the pre- or posttreatment scan. Forty eyes of 26 patients successfully completed the study with the 6 × 6 mm scan protocol, while 9 eyes of 6 patients successfully completed the study with the 3 × 3 mm scan protocol, with sufficiently high pre- and posttreatment OCTA image quality. All patients who completed the study with the 3 × 3 mm scan protocol also completed the study with the 6 × 6 mm scan protocol. Demographic data for patients who successfully completed the study using the 6 × 6 or 3 × 3 mm scan protocol are shown in Table 1.

All patients who successfully completed the study received 3 intravitreal monthly bevacizumab injections. Intraoperative and postoperative complications associated with the injections included mild subconjunctival hemorrhage or transient floaters in some cases, with no major complications related to injections occurring in the study.

### 3.2. Quantitative Analysis and Comparison of Pretreatment and Posttreatment Data

Table 2 summarizes the quantitative analysis of the pre- and posttreatment results of the 6 × 6 mm scan group, while Table 3 summarizes the quantitative analysis of the pre- and posttreatment results of the 3 × 3 mm scan group.

**Figure 4:**
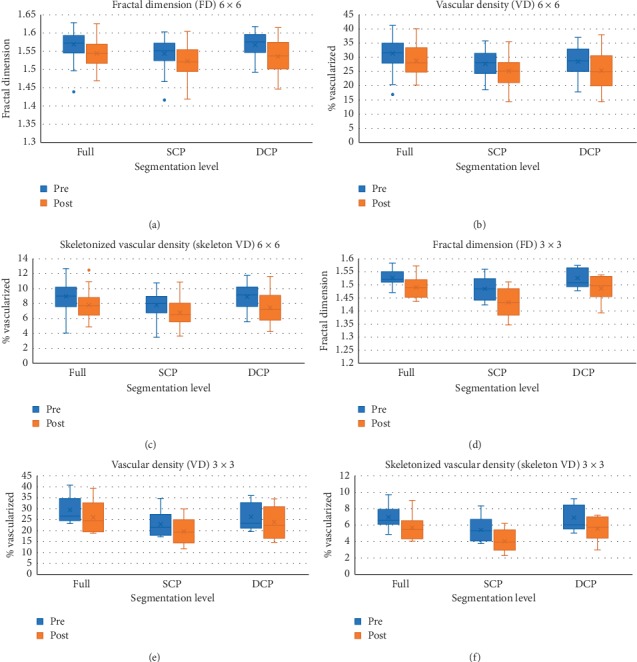
Boxplots of pre- and posttreatment values from both the 6 × 6 and 3 × 3 mm scan protocols at different segmentation levels. In the 6 × 6 mm scan group, there was a statistically significant decrease in the FD, VD, and skeleton VD of Full, SCP, and DCP (a, b, and c). In the 3 × 3 mm scan group, there was a statistically significant decrease in the FD and VD-Full and SCP and the skeleton VD-SCP. There was a decrease in skeleton VD-Full and FD, VD, and skeleton VD-DCP but these were not statistically significant (d, e, and f).

In the 6 × 6 mm scan group, there was a statistically significant increase in the size of the FAZ area and a statistically significant decrease in the FD, VD, and skeleton VD of Full, SCP, and DCP (Table 2, Figure 4).

In the 3 × 3 mm scan group, there was an increase in the size of the FAZ area, but this was not statistically significant. There was a statistically significant decrease in the FD and VD-Full and SCP and in the skeleton VD-SCP (Figure 4). There was a decrease in skeleton VD-Full and FD, VD, and skeleton VD-DCP but these were not statistically significant (Table 3; Figure 4).

### 3.3. Correlations between Baseline Data (6 × 6 Protocol Only)

In the 6 × 6 group, pretreatment logMAR CDVA significantly positively correlated with pretreatment CMT (*r* = 0.617, *p* < 0.001), pretreatment parafoveal thickness (*r* = 0.660, *p* < 0.001), and severity of retinopathy (*r* = 0.357, *p*=0.024). The severity of retinopathy positively correlated with pretreatment CMT (*r* = 0.354, *p*=0.025) but negatively correlated with pretreatment FD-SCP (*r* = −0.353, *p*=0.026), pretreatment FD-DCP (*r* = −0.378, *p*=0.016), pretreatment VD-DCP (*r* = −0.315, *p*=0.048), pretreatment skeleton VD-SCP (*r* = −0.35, *p*=0.027), and pretreatment skeleton VD-DCP (*r* = −0.40, *p*=0.011).

### 3.4. Factors Associated with Posttreatment Changes in Fractal Dimension (6 × 6 Protocol Only)

**Figure 5:**
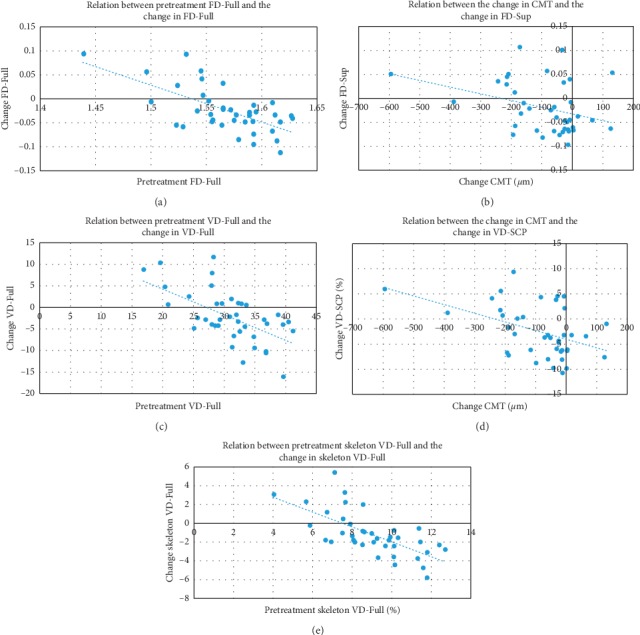
Examples of scatter plots for factors significantly associated with posttreatment changes using univariate analysis. There was a significant negative correlation between (a) pretreatment FD-Full and the change in FD-Full (*r* = −0.541, *p* < 0.001), (b) the change in CMT and the change in FD-SCP (*r* = −0.324, *p*=0.042), (c) pretreatment VD-Full and the change in VD-Full (*r* = −0.560, *p* < 0.001), (d) the change in CMT and the change in VD-SCP (*r* = −0.389, *p*=0.013), and (e) pretreatment skeleton VD-Full and the change in skeleton VD-Full (*r* = −0.694, *p* < 0.001).

Using univariate analysis with Spearman's rank test, there was a statistically significant negative correlation between pretreatment FD-Full and the change in FD-Full (*r* = −0.541, *p* < 0.001) (Figure 5(a)), pretreatment FD-SCP and the change in FD-SCP (*r* = −0.546, *p* < 0.001), the change in CMT and the change in FD-SCP (*r* = −0.324, *p* = 0.042) (Figure 5(b)), and pretreatment FD-DCP and the change in FD-DCP (*r* = −0.345, *p* = 0.029). The severity of retinopathy at baseline positively correlated with the change in FD-DCP only (*r* = 0.345, *p* = 0.029).

Using multivariate analysis, there was a significant negative correlation between pretreatment FD-Full and the change in FD-Full (*p* < 0.001), pretreatment FAZ area and the change in FD-Full (*p*=0.039), pretreatment FD-SCP and the change in FD-SCP (*p* < 0.001), pretreatment FD-DCP and the change in FD-DCP (*p* < 0.001), and pretreatment FAZ area and the change in FD-DCP (*p*=0.044).

### 3.5. Factors Associated with Posttreatment Changes in Vascular Density (6 × 6 Protocol Only)

Using univariate analysis with Spearman's rank test, there was a statistically significant negative correlation between pretreatment VD-Full and the change in VD-Full (*r* = −0.560, *p* < 0.001) (Figure 5(c)), pretreatment VD-SCP and the change in VD-SCP (*r* = −0.497, *p* = 0.001), the change in CMT and the change in VD-SCP (*r* = −0.389, *p* = 0.013) (Figure 5(d)), and pretreatment VD-DCP and the change in VD-DCP (*r* = −0.441, *p* = 0.004). The CMT at baseline positively correlated with the change in VD-SCP only (*r* = 0.319, *p* = 0.045).

Using multivariate analysis, there was a significant negative correlation between pretreatment VD-Full and the change in VD-Full (*p* < 0.001), pretreatment FAZ area and the change in VD-Full (*p* = 0.017), pretreatment skeleton VD-SCP and the change in VD-SCP (*p* < 0.001), hypertension and the change in VD-SCP (*p* = 0.001), type of DM and the change in VD-SCP (*p* = 0.01), pretreatment FAZ area and the change in VD-SCP (*p* = 0.005), and pretreatment FD-DCP and the change in VD-DCP (*p* = 0.001)(*p* = 0.001).

### 3.6. Factors Associated with Posttreatment Changes in Skeleton Vascular Density (6 × 6 Protocol Only)

Using univariate analysis with Spearman's rank test, there was a statistically significant negative correlation between pretreatment skeleton VD-Full and the change in skeleton VD-Full (*r* = −0.694, *p* < 0.001) (Figure 5(e)), pretreatment skeleton VD-SCP and the change in skeleton VD-SCP (*r* = −0.667, *p* < 0.001), the change in CMT and the change in skeleton VD-SCP (*r* = −0.377, *p* = 0.016), and pretreatment skeleton VD-DCP and the change in skeleton VD-DCP (*r* = −0.508, *p* = 0.001). The severity of retinopathy at baseline positively correlated with the change in skeleton VD-DCP only (*r* = 0.365, *p* = 0.021).

Using multivariate analysis, there was a significant negative correlation between pretreatment skeleton VD-Full and the change in skeleton VD-Full (*p* < 0.001), pretreatment skeleton VD-SCP and the change in skeleton VD-SCP (*p* < 0.001), and pretreatment FD-DCP and the change in skeleton VD-DCP (*p* < 0.001).

## 4. Discussion

Our study was a prospective interventional case series done to evaluate the effect of repeated intravitreal anti-VEGF injections on the macular perfusion of diabetic patients with DME using OCTA. A total of 40 eyes of 26 patients were successfully imaged before and after 3 monthly intravitreal anti-VEGF injections using the 6 × 6 mm macular scan protocol of OCTA, while 9 eyes of 6 patients were imaged using the 3 × 3 mm macular scan protocol. All patients were treatment naïve at the beginning of the study and were treated with 3 intravitreal bevacizumab injections for DME during the study.

Using the 6 × 6 mm scan protocol, there were statistically significant changes in all parameters measured possibly indicating a worsening of macular perfusion associated with intravitreal injections, where we found an 8.1% increase in the mean size of the FAZ area, a 1.3% decrease in the mean FD-Full and mean FD-SCP, a 1.9% decrease in the mean FD-DCP, an 8% decrease in the mean VD-Full, a 9.1% decrease in the mean VD-SCP, a 10.6% decrease in the mean VD-DCP, a 13.3% decrease in the mean skeleton VD-Full, a 12.5% decrease in the mean skeleton VD-SCP, and a 16.3% decrease in the mean skeleton VD-DCP following 3 months of intravitreal bevacizumab injections. We found that the changes in the FD values were the smallest, while changes in the skeleton VD values were the largest indicating that skeleton VD may be the most sensitive parameter for measuring retinal perfusion changes as previously reported [20].

Findings in the 3 × 3 mm scan protocol mirrored those in the 6 × 6 mm scan protocol but were more pronounced, possibly indicating more affection of the parafoveal vascularity compared to the perifoveal vascularity in association with intravitreal injections. We found a 2.6% decrease in the mean FD-Full, a 3.4% decrease in the mean FD-SCP, an 11.5% decrease in the mean VD-Full, a 14.3% decrease in the mean VD-SCP, and a 25.1% decrease in the mean skeleton VD-SCP following 3 monthly intravitreal bevacizumab injections that were all statistically significant. There were also an 8.3% increase in the mean size of the FAZ area, a 2.6% decrease in the mean FD-DCP, an 8.8% decrease in the mean VD-DCP, an 18.5% decrease in the mean skeleton VD-Full, and a 19.7% decrease in the mean skeleton VD-DCP. These changes, however, were not statistically significant, possibly due to the smaller number of eyes imaged using the 3 × 3 mm protocol.

Several previous studies have been performed to evaluate the effect of repeated intravitreal anti-VEGF injections on the macular perfusion in diabetic patients using fluorescein angiography. In the BOLT study, a prospective study that compared the efficacy of 6 weekly intravitreal bevacizumab injections to 4-monthly modified ETDRS macular laser treatment, there was no evidence of worsening of macular ischemia in either group at 4 months after treatment initiation. A total of 40 eyes were evaluated in the bevacizumab group. This study, however, used fluorescein angiography to analyze changes in the macular perfusion, evaluated changes only related to the fovea, and assessed the status of perifoveal capillaries qualitatively using human graders [14].

In an analysis of the RIDE and RISE studies, 2 parallel prospective multicenter trials comparing 2 doses of ranibizumab (0.3 and 0.5 mg) to sham injections, there was worsening of posterior retinal nonperfusion in all groups but more in the sham injected group. The authors concluded that monthly injections of ranibizumab can thus slow, but not completely prevent, retinal capillary closure in patients with DME. Again, this study depended on FA images for the pre- and posttreatment analyses and used trained human graders [15]. In this study, the authors hypothesized that worsening of retinal nonperfusion in patients with DME could be the result of VEGF-induced leukostasis that was partially reversed by the anti-VEGF therapy [15, 21]. In an electron microscopical investigation of retinal capillaries in VEGF-A-induced retinopathy in monkeys, however, there was no leukocytes adherent to the vascular wall [22]. This study, however, identified endothelial cell hypertrophy that is induced by local VEGF-A production as another possible cause of VEGF-induced worsening of capillary nonperfusion by causing progressive luminal narrowing [22].

In another study analyzing the effect of aflibercept for DME on the macular perfusion (analysis of VISTA patients), there was both more improvement in retinal nonperfusion and slowing of worsening retinal nonperfusion with aflibercept compared to laser treatment [16]. This study, however, also used fluorescein angiography and human graders.

OCTA is a new noninvasive modality that is capable of resolving the different retinal vascular layers separately to the capillary level at a higher resolution compared to conventional FA [17, 23, 24]. It has the ability to precisely and reliably delineate areas of capillary dropout and to image the FAZ without obscuration by dye leakage compared to FA [23, 24]. It allows quantitative measurements of the vascular density and fractal dimension reliably and reproducibly in the macular area in an objective manner that does not require human graders [20, 25, 26]. Furthermore, unlike FA, OCTA does not require intravenous dye injection which has been associated with side effects including nausea, vomiting, and anaphylaxis [27]. This allows OCTA to be repeated frequently with no risks or discomfort to the patient. These advantages may allow OCTA to be more suited for the analysis of changes in macular perfusion following DME treatment [28].

In a study investigating the effect of aflibercept injections for treatment naïve wet age-related macular degeneration on the macular vascular density using OCTA, there was a statistically significant decrease in the superficial foveal and parafoveal vascular density after 1, 2, and 3 injections compared to baseline (approximately 31% reduction in foveal vascular density and 6.6% reduction in parafoveal vascular density after 3 injections) [29]. This is interesting since patients with a history of retinal vascular disease were excluded from the study which suggests that anti-VEGF injections may affect the vascular density of normal retinal vessels. The automated measurements of the AngioVue software were used in this study and the authors hypothesized that aflibercept may result in decreased vascular density as measured by OCTA due to either decrease in nitric oxide, leading to vasoconstriction, or capillary rarefaction, which was described in experimental mouse models treated with VEGF inhibitor [29, 30]. Limitation of this study included the small number of included eyes (15 eyes) and the lack of a control group.

OCTA was also used to evaluate the effect of a single anti-VEGF injection on the macular perfusion in patients with macular edema secondary to DR or central retinal vein occlusion (CRVO) in a prospective noncomparative case series [31]. This study found that the FAZ area and the foveal and parafoveal vessel density of the SCP and DCP did not significantly change after the single injection. Further analysis of data from the study, however, revealed an increase in the size of the FAZ and a decrease in the vascular density of the foveal area following the single injection which, however, was not statistically significant. This may be due to the relatively small number of eyes included in the study (18 eyes), the variability in the anti-VEGF agent used (bevacizumab in 14 eyes, aflibercept in 3 eyes, and ranibizumab in 1 eye), the inclusion of 2 different causes of macular edema (DME in 13 eyes and CRVO in 5 eyes), the short duration of treatment (1 month), and the use of vascular density measurements from the machine software. The study also did not exclude patients with a history of previous anti-VEGF injections which may have influenced the results.

In another retrospective study evaluating the effect of repeated intravitreal anti-VEGF injections for treatment of DME or PDR on the macular perfusion using OCTA 3 × 3 mm and 6 × 6 mm scan protocols, there was no statistically significant difference in the macular perfusion after 1, 2, and 3 injections using both scanning protocols [32]. Although the study included 46 eyes which performed OCTA after the first injection, only 28 and 26 eyes performed OCTA after the 2^nd^ and 3^rd^ injections, respectively. Unlike our study, the type of anti-VEGF agent used was variable (45.7% bevacizumab, 42.4% aflibercept, and 11.9% ranibizumab) and the mean interval between injections was 47 days. Half of the eyes (23 eyes) that performed OCTA after the 1^st^ injection were not treatment naïve. Quantification of the vascular density was performed automatically using the proprietary built-in machine software (AngioVue software) using the ETDRS grid.

Commercially available OCTA is currently limited to evaluating the perfusion of the macular area but the technology is rapidly evolving. Using ultrawide field imaging, anti-VEGF injections were found to be associated with improved DR severity score (DRSS) on ultrawide field color fundus photographs but with no evidence of reperfusion of either arterioles or venules in or around nonperfusion areas on ultrawide field FA following 3 monthly injections [33]. On the contrary, following the last injection, 83% of studied eyes showed occlusion of few vessel segments (mean: 6 ± 11 per eye) passing through the nonperfusion areas. Interestingly, similar to our study, this study also used i2k Align retina software to align pre- and posttreatment images allowing only areas common to both images to be compared. In another study performed by the same group of investigators in which ultrawide field imaging was done using both swept-source widefield OCTA and ultra-widefield FA, there was no improvement detected in retinal nonperfusion nor evidence of arteriolar or venular reperfusion using either modality following 3 intravitreal monthly injections of anti-VEGF agents despite an improvement in the DRSS. There was, however, occlusion of some distal vessel segments following the injections, a mean of 2.33 and 3.7 newly occluded vessels per eye on ultra-widefield FA and widefield OCTA, respectively. The study only included 10 eyes of 9 patients, however, and used both ranibizumab and aflibercept [34].

Choroidal thickness is best evaluated using enhanced depth imaging (EDI) OCT which can also be used to monitor changes in the choroidal thickness during treatment and follow-up of retinal diseases [35]. Changes in the choroidal thickness following anti-VEGF injections for DME have been previously analyzed in several studies, with one study showing a decrease in the central choroidal thickness after repeated anti-VEGF injections, which was not, however, associated with either changes in visual acuity or foveal thickness [36]. The authors therefore recommended a cautious approach when using aggressive anti-VEGF therapy for DME. Correlation of VD changes, including choroidal VD changes, obtained using OCTA to choroidal thickness changes detected on EDI-OCT following treatment for DME may provide useful information.

An important factor that could influence the measurements of vascular density using OCTA is the scan signal strength index (SSI). In a study of the effect of signal strength on OCTA metrics, differences in signal strength were associated with statistically significant differences in measurements of vascular density, perfusion density, and FAZ metrics with increased signal strength being associated with a significantly increased value of all measurements [37]. Consideration of this data is essential for correct analysis of OCTA data. In our study, there was no significant difference between pretreatment and posttreatment imaging signal strength, which further supports the validity of our results.

Another factor that could also influence OCTA measurements is IOP. In a study evaluating macular and peripapillary vascular density changes immediately following an intravitreal anti-VEGF injection for various pathologies, there was a statistically significant decrease in angiographic perfusion density in most areas of the superficial and deep layer macular vascular density, with more affection of the superficial layer, and the overall optic nerve head and the radial peripapillary capillary layer, preferentially temporal [38]. In that study, the mean pretreatment IOP was 17.15 mmHg, while the mean immediate posttreatment IOP, taken approximately 15 seconds after the injection, was 46.35 mmHg as measured by Tonopen (Reichert, Depew, NY). The superficial macular density decreased by 7.8%, while the deep macular density decreased by 3.5% immediately (within 3 minutes) following the injection. The authors hypothesized that the acute rise in IOP was the cause of reduced vascular density observed in the study. In our study, there was no statistically significant difference between pretreatment and posttreatment IOP suggesting that IOP did not have a significant effect on our measurements.

An increase in the FAZ size following intravitreal bevacizumab injections for DME has also been previously reported using FA in several noncomparative studies [39, 40]. In one of these studies, there was a 19.7% increase in the FAZ area 6–8 weeks following a single bevacizumab injection for DME [39], while another study showed a 13% increase in the FAZ area following 3 monthly bevacizumab injections which was greater in patients with milder DR [40]. This was in agreement with our results and could be due to progression of ischemia due to the underlying DR or due to VEGF inhibition or both [40]. Controlled studies are needed to further explain these findings.

Several explanations could account for our findings. First, although long-term treatment with anti-VEGF agents has been shown to improve the DRSS, studies have shown that they are not able to completely halt the progressive retinal capillary closure associated with DR but to only slow it down compared to sham or laser [15, 16, 33]. This was consistent with our findings where we found a significant decrease in retinal vascular density associated with anti-VEGF injections, however, whether this decrease could have been more in the absence of injections or using other treatment modalities could not be assessed in our study due to the absence of a control group. Second, in a study using Doppler ultrasonography, there was a statistically significant decrease of 10%, 20%, and 20% in the mean blood flow velocity of the central retinal artery, temporal posterior ciliary arteries, and ophthalmic arteries, respectively, 4 weeks following a single intravitreal bevacizumab injection in patients with wet age-related macular degeneration [41]. The authors concluded that bevacizumab may result in hypoperfusion of the whole globe through vasoconstriction and decrease in capillary density. This could explain the decreased vascular density and flow detected by OCTA following bevacizumab injections in our study. Third, in a recent study of eyes with DME, both ranibizumab and bevacizumab injections were found to result in a significant constriction in the retinal blood vessels diameter measured using a semiautomated system [42]. In that study, following a single injection of treatment naïve eyes, the central retinal artery diameter decreased by 4.5% and the central retinal vein diameter decreased by 7.8% in the ranibizumab group, while the central retinal artery diameter decreased by 2.9% and the central retinal vein diameter decreased by 3.2% in the bevacizumab group one month after the injection. There was no change in the vessel diameter in the untreated control group. This effect may be due to inhibition of nitric oxide following VEGF inhibition which results in vasoconstriction and possible decrease in retinal blood flow which may explain the decreased vascular density and flow observed following bevacizumab injections in our study. Fourth, VEGF-A plays an important role in both physiological vascular development and pathological neovascularization in the retina and choroid, and the retinal vessels are initially dependent on VEGF as a survival factor [43], but such dependence is lost as soon as capillaries are covered by pericytes [44, 45]. In DR, there is early loss of pericytes [46], and this may perhaps render capillary endothelial cells in patients with DR susceptible to anti-VEGF inhibition [47], leading to endothelial apoptosis and decreased vascular density.

Using univariate and multivariate analysis, we have found that pretreatment FD, VD, and skeleton VD at each capillary layer significantly negatively correlate with the change in FD, VD, and skeleton VD at the corresponding capillary layer, respectively. This may be due to a floor effect, where eyes with more ischemia show less change over time due to a lower baseline vascularity as previously suggested [40].

This study is the largest prospective study to date evaluating the effect of 3 intravitreal monthly bevacizumab injections on the macular perfusion of diabetic patients with DME using OCTA. Strengths of the study include the prospective nature of the study with predefined study objectives, the strict exclusion criteria including previous treatment for macular edema, the inclusion of patients with a single diagnosis (DME), the use of a single type of anti-VEGF agent, the automated alignment of pre- and posttreatment OCTA images, the use of a previously validated and customized image processing technique, and the previously unreported analysis of posttreatment changes in fractal dimension, a measure of complexity and branching of retinal vessels, and skeleton vascular density, which is a more sensitive approach for estimation of retinal nonperfusion [20]. Limitations of our study include the relatively small number of patients, inclusion of both eyes of some patients, the relatively short period of treatment, and the absence of a control group. Having a proper control group, however, could be difficult, as most patients with DME benefit from, and are currently being treated at least initially with, anti-VEGF agents. In the light of our findings, however, having a sham control group in a future study may seem proper for at least a short-term period. Several imaging artifacts are currently associated with OCTA imaging and may have influenced our results [48, 49]; however, we have taken several measures to limit the effects of these artifacts on the results of our study by excluding patients with media opacities, repeating scans until no major obvious artifacts were present in the image, excluding patients with persisting major artifacts, including only images with an SSI higher than 5, manually correcting minor segmentation errors, using full thickness retinal slabs which are less affected by edema and segmentation errors, and using complex thresholding and filtering processes to create the final binarized images.

## 5. Conclusion

In conclusion, our study showed that, using OCTA, there was a significant increase in the FAZ area and a significant decrease in the fractal dimension and vascular density associated with three monthly intravitreal bevacizumab injections for the treatment of DME, which may warrant caution while using anti-VEGF agents for the long-term treatment of diabetic patients. The significance of this, however, is not totally clear since our patients still showed significant improvement in CDVA and CMT with only changes in the SCP showing a significant negative correlation with changes in CMT. Whether these vascular changes are irreversible or if they result in long-term visual consequences is not known especially that these patients may require prolonged periods of treatment. Several possible mechanisms could explain these vascular changes but longer-term studies using OCTA, preferably in the form of large randomized controlled trials, are needed to further confirm and explain the findings of this study and their significance.

## Figures and Tables

**Table 1:** Demographic data of included patients in both scanning protocols.

	6 × 6 mm protocol		3 × 3 mm protocol	
Number of patients	26		6	
Males	12	46.2%	1	16.7%
Females	14	53.8%	5	83.3%

Number of eyes	40		9	
Males	19	47.5%	1	11.1%
Females	21	52.5%	8	88.9%

Age (mean ± SD), yrs	54.8 ± 6.52		51.56 ± 6.54	

DM type (patients)				
Type 1	3	11.5%	1	16.7%
Type 2	23	88.5%	5	83.3%

DM duration (mean ± SD), yrs	16.53 ± 6		12 ± 6.87	

HbA1C (mean ± SD), %	8.07 ± 1.77		8.8 ± 1.48	

DM treatment (patients)				
Oral drugs	7	26.9%	3	50%
Insulin	19	73.1%	3	50%

Hypertension (patients)	14	53.8%	2	33.3%

Retinopathy grade (eyes)				
Mild NPDR	8	20%	3	33.3%
Moderate NPDR	14	35%	3	33.3%
Severe NPDR	9	22.5%	3	33.3%
PDR	9	22.5	0	0%

Lens status (eyes)				
Nuclear sclerosis	24	60%	5	55.6%
Nuclear cataract +1	6	15%	1	11.1%
Nuclear cataract +2	1	2.5%	0	0%
Posterior subcapsular	7	17.5%	3	33.3%
Pseudophakic	2	5%	0	0%

**Table 2:** Quantitative analysis and comparison of the pre- and posttreatment results of the 6 × 6 mm scan group.

Metric	Pretreatment	Posttreatment	Change	% change	*p* value^a^
LogMAR CDVA (mean ± SD)	0.68 ± 0.34	0.47 ± 0.25	−0.21 ± 0.27	−30.9%	<0.001^b^
CMT (mean ± SD), *μ*m	422 ± 131	334 ± 98	−88 ± 132	−20.9%	<0.001^b^
PFT (mean ± SD), *μ*m	413 ± 76	364 ± 45	−49 ± 72	−11.9%	<0.001^c^
IOP (mean ± SD), mmHg	17.6 ± 3.1	16.6 ± 3.7	−0.31 ± 3.2	−1.8%	0.737^c^
Signal strength (mean ± SD)	7.13 ± 1.57	7.15 ± 1.59	0.05 ± 1.04	0.7%	0.884^c^
FAZ area (mean ± SD), mm^2^	0.37 ± 0.11	0.4 ± 0.16	0.03 ± 0.1	8.1%	0.041^c^
FD-Full (mean ± SD)	1.57 ± 0.04	1.54 ± 0.04	−0.02 ± 0.05	−1.3%	0.003^c^
FD-SCP (mean ± SD)	1.54 ± 0.04	1.52 ± 0.04	−0.02 ± 0.05	−1.3%	0.021^c^
FD-DCP (mean ± SD)	1.57 ± 0.04	1.54 ± 0.05	−0.03 ± 0.05	−1.9%	<0.001^c^
VD-Full (mean ± SD), %	31.3 ± 5.58	28.8 ± 5.18	−2.5 ± 5.72	−8%	0.01^c^
VD-SCP (mean ± SD), %	27.8 ± 4.09	25.3 ± 4.78	−2.53 ± 5.13	−9.1%	0.003^c^
VD-DCP (mean ± SD), %	28.5 ± 5.28	25.5 ± 5.96	−3.03 ± 6.24	−10.6%	0.004^c^
Skeleton VD-Full (mean ± SD), %	9 ± 1.99	7.8 ± 1.76	−1.2 ± 2.32	−13.3%	0.003^c^
Skeleton VD-SCP (mean ± SD), %	7.77 ± 1.6	6.8 ± 1.62	−0.97 ± 2.16	−12.5%	0.007^c^
Skeleton VD-DCP (mean ± SD), %	8.9 ± 1.8	7.44 ± 2.03	−1.45 ± 2.36	−16.3%	<0.001^c^

CDVA, corrected distance visual acuity; CMT, central macular thickness; DCP, deep capillary plexus; FAZ, foveal avascular zone; FD, fractal dimension; Full, full retinal thickness; IOP, intraocular pressure; LogMAR, logarithm of the minimum angle of resolution; PFT, parafoveal thickness; SCP, superficial capillary plexus; VD, vascular density. ^a^Statistically significant if <0.05. ^b^Wilcoxon signed rank test. ^c^Paired *t*-test.

**Table 3:** Quantitative analysis and comparison of the pre- and posttreatment results of the 3 × 3 mm scan group.

Metric	Pretreatment	Posttreatment	Change	% change	*p* value^a^
LogMAR CDVA (mean ± SD)	0.63 ± 0.34	0.51 ± 0.38	−0.12 ± 0.24	−19%	0.122^b^
CMT (mean ± SD), *μ*m	370 ± 69	276 ± 51	−93 ± 74	−25.1%	0.008^b^
PFT (mean ± SD), *μ*m	384 ± 34	331 ± 25	−53 ± 42	−13.8%	0.005^c^
IOP (mean ± SD), mmHg	17.8 ± 1.9	15.7 ± 2.7	−2.5 ± 2.6	−14%	0.064^c^
Signal strength (mean ± SD)	5.9 ± 1.4	6.3 ± 1.1	0.4 ± 0.9	6.8%	0.169^c^
FAZ area (mean ± SD), mm^2^	0.36 ± 0.09	0.39 ± 0.12	0.03 ± 0.04	8.3%	0.067^c^
FD-Full (mean ± SD)	1.53 ± 0.03	1.49 ± 0.04	−0.04 ± 0.05	−2.6%	0.046^c^
FD-SCP (mean ± SD)	1.48 ± 0.05	1.43 ± 0.06	−0.05 ± 0.05	−3.4%	0.01^c^
FD-DCP (mean ± SD)	1.53 ± 0.04	1.49 ± 0.05	−0.04 ± 0.06	−2.6%	0.073^c^
VD-Full (mean ± SD), %	29.5 ± 5.97	26.1 ± 7.67	−3.38 ± 4.08	−11.5%	0.038^c^
VD-SCP (mean ± SD), %	22.9 ± 6.17	19.7 ± 6.3	−3.27 ± 3.18	−14.3%	0.015^c^
VD-DCP (mean ± SD), %	26.2 ± 6.22	23.9 ± 7.32	−2.3 ± 3.95	−8.8%	0.118^c^
Skeleton VD-Full (mean ± SD), %	6.93 ± 1.4	5.65 ± 1.6	−1.28 ± 1.75	−18.5%	0.059^c^
Skeleton VD-SCP (mean ± SD), %	5.45 ± 1.6	4.09 ± 1.3	−1.37 ± 1.2	−25.1%	0.009^c^
Skeleton VD-DCP (mean ± SD), %	6.86 ± 1.6	5.52 ± 1.5	−1.35 ± 1.9	−19.7%	0.066^c^

CDVA, corrected distance visual acuity; CMT, central macular thickness; DCP, deep capillary plexus; FAZ, foveal avascular zone; FD, fractal dimension; Full, full retinal thickness; IOP, intraocular pressure; LogMAR, logarithm of the minimum angle of resolution; PFT, parafoveal thickness; SCP, superficial capillary plexus; VD, vascular density. ^a^Statistically significant if <0.05. ^b^Wilcoxon signed rank test. ^c^Paired *t*-test.

## Data Availability

The datasets used and/or analyzed during the current study are available from the corresponding author upon reasonable request.
